# Dementia and hearing loss: from risk to mechanisms and management

**DOI:** 10.3389/frdem.2026.1736003

**Published:** 2026-02-17

**Authors:** Emma E. Broome, Sian Calvert, Eithne Heffernan, Helen Henshaw, Aaliyah Khan, Vassilis Pelekanos, Joseph Sollini, Jack Stancel-Lewis, Tom Dening

**Affiliations:** 1 National Institute for Health and Care Research (NIHR), Nottingham Biomedical Research Centre, Nottingham, United Kingdom; 2 Mental Health and Clinical Neurosciences, School of Medicine, University of Nottingham, Nottingham, United Kingdom; 3 School of Sport, Exercise & Health Sciences, Loughborough University, Loughborough, United Kingdom; 4 School of Medicine, University of Nottingham, Nottingham, United Kingdom; 5 Department of Social Sciences, School of Humanities and Social Sciences, University of Nicosia, Nicosia, Cyprus; 6 Nottingham University Business School, University of Nottingham, Nottingham, United Kingdom

**Keywords:** cognitive impairment, dementia, hearing loss, mechanisms, presbycusis

## Abstract

Hearing loss in midlife is an important and potentially modifiable risk factor for the development of dementia. Research examining the association between dementia and hearing loss has expanded rapidly; however, evidence for the mechanisms linking the two conditions is inconclusive, limiting the development of targeted interventions. This review provides a critical overview of current evidence on dementia risk in relation to hearing loss, proposed mechanisms underpinning this association, and emerging evidence on the effectiveness of hearing interventions in modifying trajectories of cognitive decline, dementia risk, and disease progression. Alongside its role as a risk factor, hearing loss commonly co-occurs with dementia, highlighting the need for integrated approaches to care that address the considerable impact of these co-morbid conditions on individuals and communities. Finally, we emphasise the importance of including diverse populations in future research to improve generalisability of findings and help advance equity in dementia prevention and care.

## 1 Introduction

Clinicians working with older people have long been aware that cognitive and hearing impairments often co-occur. There has been an increase in research examining hearing loss and dementia over the last decade, stimulated by the first Lancet Commission report on dementia prevention, intervention and care (Livingston et al., 2017), which identified hearing loss from midlife onwards as the largest potentially modifiable population-attributable risk factor for dementia. From this, further research has explored the accuracy of this estimate, the possible underlying mechanisms linking hearing conditions and dementia, and the potential for hearing interventions to reduce the risk of future dementia or to modify the trajectory of cognitive decline for those at risk.

Dementia is a group of symptoms caused by different diseases that affect the brain, impacting 55 million people worldwide (World Health Organization, 2023). There is no known cure, but evidence suggests lifestyle modification can help reduce dementia risk or slow its progression (Livingston et al., 2024; Patterson, 2018; World Health Organisation, 2025). Hearing loss is defined as a reduced ability to detect sounds compared to someone with normal hearing, typically characterised by pure-tone audiometric hearing thresholds of 20dB hearing level (HL) or worse in each ear (World Health Organization, 2024). The primary management strategy for hearing loss is the provision of hearing aids or cochlear implants to provide access to sounds. It affects over 1.5 billion people globally and is a significant contributor to years lived with disability (World Health Organization, 2024; Haile et al., 2024). The greatest burden is in low- and middle-income countries, where access to hearing care remains limited (Newall, 2025). Hearing loss and dementia, both individually and in combination, reduce independence, increase social isolation, impair communication, and increase the burden of care compared to people living without the conditions (Shukla, 2020; Azevedo, 2021).

Hearing loss often goes undetected and unmanaged. In the context of an ageing population, the potential societal impact of addressing hearing loss as a potentially modifiable risk factor for dementia is substantial (Stancel-Lewis et al., 2025). Hearing health is an important consideration for public health, with implications extending beyond individual communication outcomes to healthcare systems, social care demand, and health inequalities (Stancel-Lewis et al., 2025; Parmar et al., 2025). The intersection of sensory and cognitive decline presents a growing challenge for healthcare systems. This review contributes to the broader field of ageing research by summarising the evidence linking dementia and hearing loss and providing an overview of key issues. We do so by asking: (1) how much dementia risk is attributable to hearing loss? (2) if hearing loss and dementia are linked, what mechanisms could explain this connection? and (3) can hearing interventions modify dementia risk and/or the trajectory of cognitive decline? We then explore areas of current and future research as they relate to the care and management of people with coexisting dementia and hearing loss.

## 2 Dementia risk and hearing loss

### 2.1 The Lancet commission on dementia

The Lancet Standing Commission on Dementia prevention, intervention, and care is an expert panel that provides ongoing, evidence-based recommendations to prevent, manage, and improve care for dementia worldwide. The (Livingston et al., 2017, 2020, 2024) Lancet Commission reports identify potentially modifiable risk factors for the development of dementia. In these models, potential risk factors are calculated using a weighted population attributable fraction (PAF) with an assumption of causation. It suggests how exposure (or non-exposure) to factors such as hearing loss, contributes to the overall incidence of dementia, based on the contributing factor’s relative risk and prevalence in a population (Mansournia and Altman, 2018). First, the magnitude of each risk factor is gained from worldwide estimates, then a sample population is used to estimate how these factors cluster within individuals (accepting that individuals often experience multiple risk factors). The PAF is influenced by the sample population data from which it is generated.

The (Livingston et al., 2017, 2020) reports included three observational studies (Deal, 2017; Lin et al., 2011; Gallacher et al., 2012) that assessed the association between hearing loss and dementia in a meta-analysis to calculate the relative PAF posed by hearing loss. In the 2017 report (Livingston et al., 2017), three key outcomes from each study were combined using a random effects model, producing a risk ratio (RR) of 1·94 [95% confidence interval (CI) 1·38–2·73], suggesting that people with hearing loss were almost twice as likely to develop dementia than those without. When multiple risk factors are combined within a model (as in the Lancet Commission), the PAF is weighted to account for the communality of risk. The PAF of a single risk factor will vary depending on the number of risk factors included in the calculations. Thus, the PAF for hearing loss has decreased from 9·1% in 2017 (Livingston et al., 2017) to 8·2% in 2020 (Livingston et al., 2020), following the inclusion of three additional risk factors: traumatic brain injury, alcohol consumption and pollution. It is important to note that PAFs for dementia are calculated using data primarily from high-income countries, yet evidence suggests that the burden of modifiable risk factors could be even greater in low-middle income countries (Paradela et al., 2024).

The 2024 Commission (Livingston et al., 2024) included data from six observational studies (four new studies were added; Brenowitz et al., 2019; Marinelli et al., 2022; Osler et al., 2019; Powell et al., 2022) and one was removed (Gallacher et al., 2012) from the analysis due to updated inclusion/exclusion criteria, with the model producing a hazard ratio of 1·37 (95% CI, 1.38–2.73). This model suggests that people with hearing loss had a 37% increased risk of developing dementia compared to those without hearing loss. Results included in the model were unadjusted for hearing intervention use as treat hearing device use as part of the causal pathway between hearing loss and dementia rather than as a confounder. The PAF for hearing loss in this model was 7%, with the inclusion of two further risk factors: vision loss and high LDL cholesterol (Livingston et al., 2024).

### 2.2 Citing the evidence from the Lancet commission models

A challenge for the field lies in how the contribution of hearing loss to dementia risk, reported by the Lancet Commission, is cited within the health research literature. Frequently, hearing loss is reported to be the ‘largest’ or ‘leading’ risk factor for the development of dementia, whilst ~93% of risk is attributable to age-related changes, genetics, or a combination of other *potentially* modifiable risk factors. When citing the report outcomes, care should be taken to interpret the findings in the context of the available evidence, and inherent model and risk factor assumptions, situated within the wider context of age-related changes and genetic risk.

The PAF suggests that hearing loss contributes to the overall incidence of dementia in terms of the *relative risk* posed together with the *prevalence* of hearing loss within a population (Mansournia and Altman, 2018). Hearing loss is highly prevalent, which influences the ‘risk’. However, claims that treating hearing loss can ‘reduce dementia cases by 7%’ probably arise from two assumptions that are not currently supported by the published evidence. First, that hearing loss *can cause* dementia—however, evidence to date suggests an association, but does not confirm causation. Second, it implies that hearing loss can be fully corrected through hearing interventions (e.g., hearing aids)—which is not the case as interventions help manage hearing loss but do not restore it.

Finally, PAF is context specific, dependent on the data on which it is modelled and can only provide estimates of risk using the best data available at that time. The PAF modelled by the Lancet Commission in 2024 includes clustering of risk estimates from a Norwegian sample, which, despite being the best available evidence, is not wholly representative of other populations. Notwithstanding, risk clustering influences the magnitude of risk factor estimates. Thus, findings should be interpreted within the broader context of model assumptions and existing evidence. More recently, Ishak et al. (2025) refined the population-attributable fraction (PAF) for dementia due to hearing loss using pooled international data, suggesting the overall contribution of hearing loss to dementia risk may be smaller but remains among the most significant potentially modifiable risk factors.

Clear and responsible communication of the association between hearing loss and dementia is essential. Messaging that emphasises the broader benefits of addressing hearing loss has been recommended in a position statement by the British Society of Audiology (British Society of Audiology, 2024). This includes guidance that hearing devices should not be recommended or marketed based on a dementia risk reduction narrative, but that addressing hearing loss is beneficial for overall wellbeing and quality of life across many areas, including cognition. Overstating the impact of hearing interventions on dementia prevention could promote unrealistic expectations. Directly linking dementia risk to hearing loss could exacerbate the stigma of hearing loss which may inadvertently discourage help-seeking (Blustein et al., 2023; Dawes and Munro, 2024). Nonetheless, the strength of the association between hearing loss and cognitive decline should not be overlooked. A recent meta-analysis (*n* = 1,548,754) found that adult-onset hearing loss significantly increases the risk of cognitive impairment (Yu, 2024), highlighting the importance of understanding how these conditions are related.

## 3 Mechanisms for the link between hearing loss and cognitive decline

**Figure 1:**
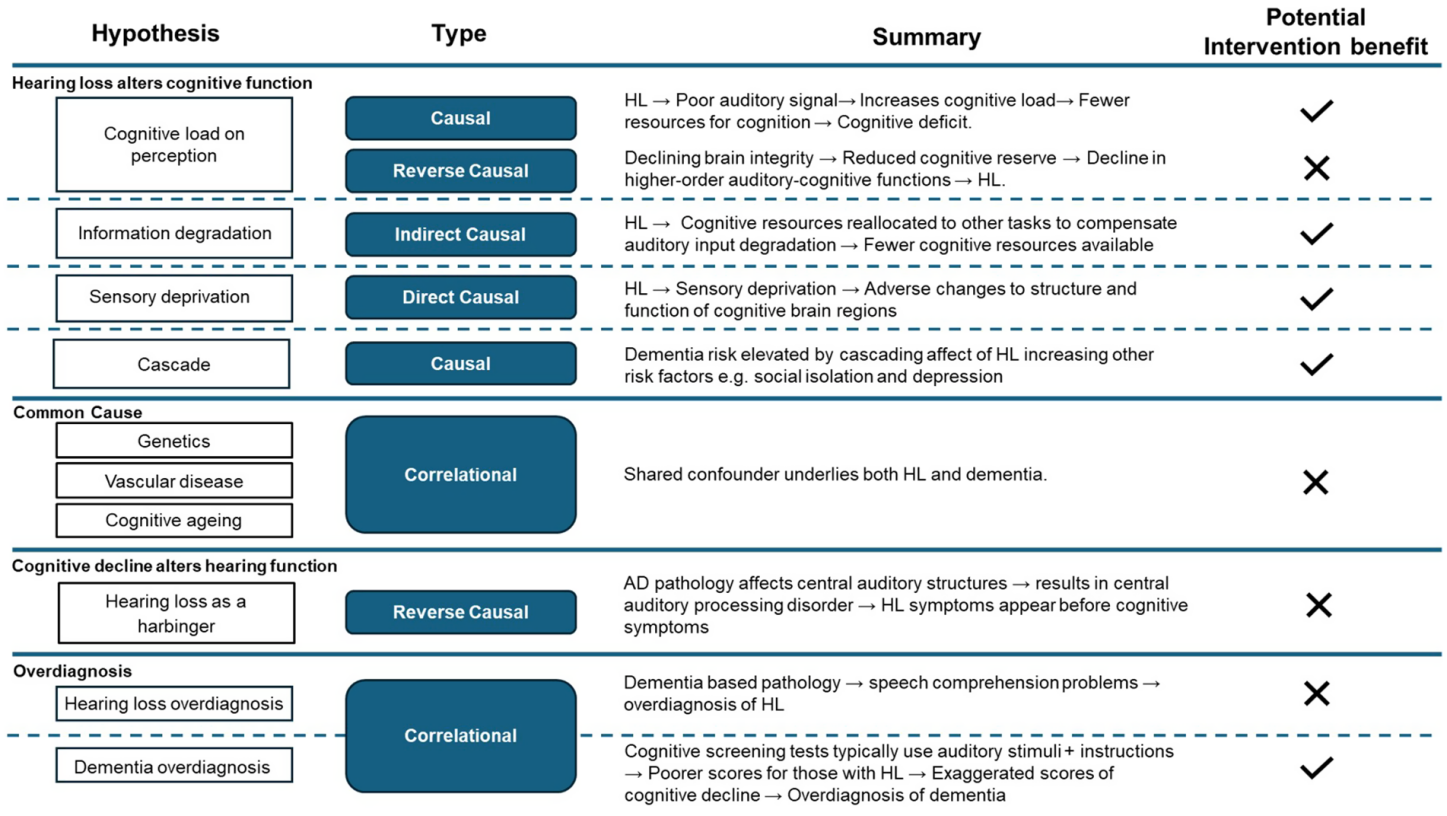
Summary of proposed hypotheses and mechanistic type. Checkmarks indicate the potential benefit of hearing intervention for dementia risk reduction/delay based on the assumption that each hypothesis occurs in isolation (although they could work in combination). HL, hearing loss; AD, Alzheimer’s disease.

Several hypotheses have been proposed to account for the observed relationship between hearing loss in midlife and the increased incidence of dementia (see Figure 1).

The plausibility and relevance of the proposed mechanisms may depend in part on the timing of hearing loss across the life course. Much of the evidence has focused on hearing loss in mid-life as a risk factor for dementia. Hearing loss in mid-life may have long-term effects on cognitive reserve, brain structure and social engagement which may increase vulnerability to neurodegeneration later in life (Livingston et al., 2017; Livingston et al., 2020; Livingston et al., 2024; Lin et al., 2011). In contrast, hearing loss occurring in later life may in some cases be a consequence of prodromal dementia-related pathology (Hardy et al., 2016; Griffiths et al., 2020). Distinguishing between these pathways is essential when interpreting mechanistic evidence and evaluating the potential impact of hearing interventions.

Proposed causal mechanisms variously suggest that: (i) hearing loss directly elevates dementia risk; (ii) hearing loss causes knock-on changes that indirectly elevate the risk; or (iii) a combination of the two. Additionally, dementia may cause an elevated risk of hearing loss diagnosis as the early brain changes related to dementia could contribute to hearing loss which is referred to as a “reverse causal” mechanism. This area is evolving, and within the literature, some interpretations of the proposed mechanisms overlap or are used interchangeably.

### 3.1 Hypothesised mechanisms

#### 3.1.1 Hearing loss alters cognitive function

##### 3.1.1.1 Cognitive load on perception

Cognitive load relates to brain activity that is consumed, or redirected, in response to a given task, to maintain task execution (Pichora-Fuller et al., 2016).

The first explanation is that degraded auditory input (peripheral hearing) demands more cognitive resources for processing, thus reducing central cognitive capacity resulting in cognitive deficits (“outside-in”) Huang and Lin, 2024). As hearing loss compromises the auditory signal, mental efforts are redirected from other tasks towards effortful listening (Uchida et al., 2019). Impairment-simulation studies in cognitively healthy young adults support this by showing reduced cognitive test performance (Füllgrabe, 2020). Real-time measures, such as pupillometry, a physiological index for cognitive load, provide a more direct assessment of how degraded speech intelligibility affects cognition (Van Engen and McLaughlin, 2018). Pupillary response measures increase for individuals with hearing loss, across a broad range of signal-to-noise ratios, compared to a narrower range for those with normal hearing (Ohlenforst et al., 2017). Although hearing loss can increase the ‘cognitive load’, and hinder task performance, there is limited explanation as to how this can lead to long-term cognitive impairment.

Second, is the concept of reserve, a backup-system compensating for brain insults and damage that would otherwise cause cognitive impairment (Stern et al., 2019). This ‘cognitive load’ hypothesis suggests an “inside-out” mechanism, where the central cognitive reserve is the limiting factor and so when peripheral demand increases, central deficits become more apparent in other words central limits affect hearing processing (Wayne and Johnsrude, 2015).

##### 3.1.1.2 Information degradation

This hypothesis builds upon the “inside-out” mechanism. Compensating for auditory input degradation may hinder higher-level cognition in favour of auditory processing and speech perception. Given the limited pool of cognitive resources allocated to different mental tasks (Tun et al., 2009), increased listening effort compromises higher-level processing, which can manifest as cognitive decline. Interventions to improve auditory processing (e.g., hearing aids), could free resources and improve higher-level cognition (Wayne and Johnsrude, 2015; Pichora-Fuller, 2003; Lin and Albert, 2014; Panza et al., 2015; Ralli et al., 2019; Slade et al., 2020).

##### 3.1.1.3 Sensory deprivation

Prolonged sensory deprivation, due to hearing loss, leads to adverse changes to the structure and function of cognitive brain regions. One possibility is that alterations in auditory system physiology and function, which has anatomical and functional connectivity with the medial temporal lobe (MTL), could cause direct changes in its function and physiology. For example, people with hearing loss have reduced temporal lobe volume (Lin and Albert, 2014; Armstrong et al., 2019; Lin et al., 2014; Wang et al., 2016; Xu et al., 2019; Zhang et al., 2022), altered resting-state functional connectivity (Loughrey et al., 2018) and a range of other physiological changes. It might also be that hearing loss causes changes in cognitive brain regions other than the MTL, which alter cognition and increase dementia risk (Powell et al., 2022).

This hypothesis generally considers hearing loss to be the primary cause of cognitive decline, but in many interpretations, it also proposes that hearing loss has consequences, such as social isolation or increased sensory load, that may indirectly increase dementia risk.

##### 3.1.1.4 Cascade

The sensory deprivation hypothesis is closely linked, and often used interchangeably with, the ‘cascade hypothesis’, which focuses on indirect changes caused by hearing loss. Social isolation and depression are associated with increased dementia risk (Desai et al., 2020; Penninkilampi et al., 2018). As hearing loss is associated with increased depressive symptoms (Cosh et al., 2019) and reduced social engagement (Shukla, 2020; Gopinath et al., 2012), it could contribute to dementia via these indirect factors (Shukla, 2020; Powell et al., 2022). Dementia risk is elevated directly via sensory deprivation and via the cascading effect of hearing loss increasing other risk factors.

#### 3.1.2 Common cause

This hypothesis suggests that a shared confounder may underlie both hearing loss and dementia.

##### 3.1.2.1 Genetics

Several genetic variants have been associated with both hearing loss and dementia (Kurniawan et al., 2012; Brenowitz et al., 2020; Abidin et al., 2021). Although studies have failed to find a shared genetic architecture, a pattern of overlapping gene sets, involved in similar molecular pathways, appears to contribute to both conditions. The APOE genotype itself may increase the risk of both Alzheimer’s Disease (AD) and hearing loss (Kurniawan et al., 2012). A higher Alzheimer’s disease genetic risk score (including APOE) was strongly linked to poorer speech-in-noise hearing and self-reported hearing difficulties in the UK Biobank dataset (Brenowitz et al., 2020). Finally, Mendelian randomisation studies have reported a possible causal association, with APOE acting as a mediator (Abidin et al., 2021). APOE genotype appears to differentially influence risk for dementia and hearing loss. The APOE ε4 allele, the strongest genetic risk factor for Alzheimer’s disease, has also been associated with poorer hearing performance and increased hearing difficulty, suggesting shared biological pathways such as lipid metabolism, neuroinflammation, or vascular dysfunction (Brenowitz et al., 2020; Abidin et al., 2021). In contrast, APOE ε2 is generally considered protective for Alzheimer’s disease, although its relationship with hearing outcomes is less well established (Ferretti, 2018).

##### 3.1.2.2 Vascular disease

Cerebral hypoperfusion, a proxy for decreased neuronal function, is linked to early hearing loss (Ponticorvo et al., 2019) and AD (Eisenmenger et al., 2023). Cerebral small vessel disease, investigated through proxies such as white matter hyperintensities, may precipitate neurodegeneration and has been associated with both hearing loss (Eckert, 2013) and cognitive decline (Söderlund et al., 2003). However, studies still show an association between hearing loss and dementia, even when controlling for vascular risk factors.

##### 3.1.2.3 Cognitive ageing

Ageing itself is a risk factor for both hearing loss and dementia, with incidence of both rising with age. Ageing of the central nervous system reduces brain integrity through volume loss, cortical thinning and neural connectivity changes. Temporal lobe atrophy appears to accelerate in older age, making this region vulnerable to both AD and age-related hearing loss (Lin et al., 2014). Mitochondrial dysfunction, oxidative distress and inflammation are associated with ageing and may also contribute to both hearing and cognitive declines (Srivastava, 2017).

#### 3.1.3 Cognitive decline alters hearing function

##### 3.1.3.1 Hearing loss as a harbinger

In this hypothesis, hearing loss is a harbinger of underlying dementia pathology. Neurodegenerative changes associated with AD can affect central auditory structures (central presbycusis), resulting in central auditory processing disorder, which most commonly manifests as speech-in-noise hearing impairment. Supporting evidence includes (i) post-mortem Alzheimer’s neuropathological evidence found in structures of the central auditory system (Baloyannis et al., 2009; Esiri et al., 1986; Lewis et al., 1987; Sinha et al., 1993), (ii) genetic studies showing a possible reverse causal relationship between AD and hearing loss (Golob et al., 2009) and (iii) behavioural studies where central auditory dysfunction was linked to cognitive decline (Gates, 2008; Mohammed et al., 2022).

#### 3.1.4 Overdiagnosis

The presence of one condition could create overdiagnosis of the other, given that (Broome et al., 2023).

##### 3.1.4.1 Dementia overdiagnosis

Cognitive screening tools may overlook the impact of hearing impairments on test performance. For example, the Montreal Cognitive Assessment Tool is orally administered, potentially resulting in false positive dementia diagnoses (Pye et al., 2017; Dupuis, 2015). Individuals with hearing loss may present with similar symptoms as those with dementia (e.g., functional decline, social withdrawal), because of hearing loss rather than cognitive decline (Broome et al., 2023).

##### 3.1.4.2 Hearing loss overdiagnosis

Dementia-based pathology could lead to speech comprehension problems, resulting in overdiagnosis of hearing loss. Increased problems with central auditory function could prompt individuals to seek referral to hearing healthcare services and receive a hearing loss diagnosis. This differs subtly from hearing loss as a harbinger, in that increased hearing loss diagnosis does not reflect an actual increase in the probability of hearing loss, but rather an increase in the probability of a person living with dementia having their hearing loss diagnosed relative to the general population (Powell et al., 2022).

#### 3.1.5 Physiological mechanisms

Neuroanatomical evidence from animal studies suggests multiple pathways link the auditory system with the hippocampal system and the MTL (Cenquizca and Swanson, 2007; Cheng et al., 2011; Xiao et al., 2018; Billig et al., 2022). Impaired auditory input may lead to physiological changes in structures related to higher-level cognitive functions.

Griffiths et al. (2020) proposed that degraded auditory input may lead to either increased or decreased activity associated with auditory cognitive processes in the MTL. These hypotheses are supported by the direct links between increased neuronal activity and AD pathology, and by animal models indicating that there are interactions between AD pathology and oscillatory activity in the MTL.

According to the ‘two-hit model’, hearing loss may constitute a ‘second hit’ on the brain, increasing dementia risk by adding to brain pathology, resulting from amyloid-beta accumulation, neurofibrillary tangles and microvascular disease (Uchida et al., 2019; Lin et al., 2014).

Hearing loss in older adults has been associated with higher CSF levels of tau or ptau181 (Azeem et al., 2023; Lasica et al., 2025), while variation in the metabotropic glutamate receptor gene may be a risk for hearing loss, possibly by modifying susceptibility to glutamate excitotoxicity. The glutamatergic pathway is also involved in AD (Panza et al., 2015). Increased brain atrophy and higher tau proteins in adult-onset hearing loss may lead to reduced cognitive reserve and elevated dementia risk.

Hearing loss may cause disruption and/or reduction in the integrity of white matter tracts (Panza et al., 2015; Ford et al., 2018).

Furthermore, there is evidence for alterations in neurotransmission including reduced GABA levels found in humans and animals with hearing loss (Slade et al., 2020). Noise-induced hearing loss in animal models is associated with increased density of the vesicular glutamate transporter and decreased density of the vesicular GABA transporter, along with hippocampal changes including tau hyperphosphorylation, an increase of mitochondrial area in hippocampal neurons, widened synaptic cleft, thinned postsynaptic density, and reduced mean optical density of Nissl bodies (Zhang et al., 2022; Azeem et al., 2023).

Hearing loss, induced by ear occlusion techniques, alters neuroinflammatory markers, which may lead to morphological microglial alterations, potentially causing impaired hippocampal neurogenesis (Azeem et al., 2023). Whereas hearing loss induced by cochlear ablation has been shown to decrease hippocampal synaptic proteins, resulting in hippocampal synapses becoming more vulnerable to damage (Ralli et al., 2019; Azeem et al., 2023).

Studies using animal models of AD suggest that inducing hearing loss in AD-model mice worsens neuropathological AD markers and leads to early AD-related disruptions at multiple levels, including: brainstem-level auditory dysfunction, synaptic and morphological alternations in the auditory cortex, elevated tau phosphorylation, neuroinflammation, oxidative imbalance, reduced cerebral glucose metabolism, and accelerated grey-matter reductions in multiple brain regions (Kim, 2020; Liu et al., 2020; Na, 2023; Paciello, 2021).

Furthermore, other animal models suggest that the association between hearing loss and dementia may be explained by cellular effects (e.g., oxidative stress, altered gene expression) or changes in neural circuit function (Johnson, 2021), or AD-related cochlear DNA damage (Park et al., 2024).

Finally, sex differences are notable in both dementia and hearing loss (Ferretti, 2018; Homans et al., 2017). Women have a higher overall rate of dementia diagnoses, largely driven by Alzheimer’s disease prevalence, whereas vascular dementia is more common in men, particularly at younger ages (Livingston et al., 2024). For hearing loss, while occupational hearing loss is more prevalent in men, age-related hearing loss is more prevalent and severe in adulthood among women (Haile et al., 2024). These sex-dependent patterns suggest that neurodegenerative pathways, and any modifiers of risk such as hearing loss, could interact differently in women and men. However, this is yet to be examined in detail.

Given that both dementia and hearing loss are multifaceted, it is likely that the mechanisms linking them are similarly complex, encompassing a range of interrelated biological, psychological, demographic, and social pathways. Thus, the potential benefits offered by hearing interventions would vary according to the proposed mechanism. Recent work by Levett et al. (2025) proposed a ‘cause–catalyst–consequence’ model, integrating neuroanatomical, psychosocial and neuropathological evidence to explain bidirectional links between hearing loss and dementia.

In summary, it remains unclear which mechanisms link hearing loss and dementia, whether they act in isolation or in combination, and how this influences the potential impact of interventions.

## 4 Can hearing interventions prevent dementia?

Although hearing interventions could benefit population health if they reduced or altered the trajectory of cognitive decline and dementia, this has yet to be robustly demonstrated. Table 1 summarises the literature on hearing interventions related to dementia and/or cognitive decline, organised by 1. controlled trials and 2. cohort and observational studies. All trials investigated hearing aids (HAs); however, marked methodological differences including recruitment, randomisation and the nature of the conditions, make the synthesis of results inappropriate.

**Table 1:** Review of primary evidence on hearing interventions and dementia prevention.

Study	N	Study design follow-up (years)	Intervention: hearing aids (HA), cochlear implants (CI)	Main findings	Supports hearing intervention modifying the trajectory of cognitive decline or risk of dementia?
Controlled trials					
van Hooren (2005) Netherlands	102	Non-randomised controlled trial. 1 yr.	HA	HA improved hearing acuity in those who received the intervention relative to those who did not, but no significant improvements in cognitive performance over 12 months.	No
Nguyen et al. (2017) France	51	Multicentre double-blind placebo crossover. 1 yr.	HA	For individuals with AD, HA did not slow cognitive decline.	No
Karawani et al. (2018) USA	32	Parallel randomised controlled trial 0·5 yrs	HA	HA led to improved working memory and enhanced cortical processing for speech stimuli. Improved working memory was correlated with increased cortical amplitudes.	Unclear
Nkyekyer et al. (2019) Australia	40	Randomised cross-over pilot. 0·5 yrs.	HA + auditory training	No improvement in cognition despite the use of HA and auditory training.	No
Lin (2023) USA	977	Multicentre, parallel group, unmasked, randomised controlled trial. 3 yrs.	HA	HAs were not associated with a reduced hazard of cognitive impairment. HAs reduced the rate of cognitive decline, for those with more risk factors for dementia at baseline.	Unclear
Yu (2025) UK	58	Pilot randomised controlled trial	HA	Observed 1.2 point difference in cognitive performance between the intervention and control groups at 6 months. However not statistically significant.	Unclear
Cohort and observational studies					
Deal et al. (2015) USA	253	Cross-sectional and longitudinal pilot 20 yrs.	HA	Participants who did not wear HA had the greatest cognitive decline. Slightly greater cognitive decline for participants who used HAs than normal hearing controls.	Yes
Dawes et al. (2015) USA	666	Longitudinal cohort	HA	No significant differences between HA users and non-users in cognitive, social engagement, or mental health outcomes at any time point.	No
Amieva et al. (2015) France	3,670	Prospective observational 25 yrs.	HA	Slower cognitive decline for those with HL who used HAs than those who did not. For those who used HAs, cognitive decline not significantly different from controls.	Yes
Qian et al. (2016) USA	100	Cross-sectional observational N/A	HA	HA users who had worse hearing performed significantly better on the MMSE than non-HA users. However, HA users did not perform significantly better in tests of executive function.	Unclear
Mosnier et al. (2018) France	70	Longitudinal cohort 5·5–8·5 yrs	CI	Low rate of progression to dementia, and cognitive function improved in some individuals with MCI at baseline, indicating possible positive effect.	Unclear
Maharani et al. (2018) UK	2040	Longitudinal cohort 18 yrs.	HA	Slower decline in episodic memory performance after, compared to before, HA use.	Yes
Amieva et al. (2018) France	3,777	Prospective observational 25 yrs.	HA	Increased disability and dementia risk in participants with HL but diminished in those participants using HA.	Yes
Völter et al. (2018) Germany	60	Longitudinal prospective 1 yr.	CI	CI were associated with improved cognitive performance and quality of life in adults aged 50 + years. Those with worse baseline neurocognitive performance had better outcomes.	Unclear
Mahmoudi et al. (2019) USA	114,862	Retrospective cohort 3 yrs.	HA	HA use in adults with HL was associated with a delay or prevention of AD or dementia, anxiety or depression and injurious falls.	Yes
Sarant et al. (2020) Australia	99	Longitudinal observational 1·5 yrs.	HA	No improvements in cognitive test battery scores relative to 18 months of HA use. Significant improvements in executive function.	Unclear
Glick and Sharma (2020) USA	41	Prospective observational 0·5 yrs.	HA	HA use was associated with a reversal in cross-modal reorganisation for individuals with ARHL. Cortical changes coincided with improvements in auditory perception and cognitive domains.	Yes
Tai et al. (2021) Taiwan	1,418	Longitudinal cohort mean 8·9 yrs	HA	In a subgroup analysis within the hearing-impaired half of the sample, HA use was associated with lower incidence of cognitive impairment when compared to HA non-users, but the adjusted hazard ratio (0·82) was non-significant.	Unclear
Cuoco et al. (2021) Italy	96	Prospective observational 0·5 yrs.	HA	Some aspects of cognition improved for those with mild HL, relative to HA use. For individuals with more severe HL, various aspects of cognition tended to worsen.	Unclear
Bucholc et al. (2021) USA	2,114	Longitudinal retrospective. Follow-up: Time to incident dementia, from MCI.	HA	HA use was associated with a reduced risk of conversion from MCI to all-cause dementia. Accelerated cognitive decline for individuals who did not use HA.	Yes
Naylor et al. (2022) UK	380,794	Retrospective observational 3·5–5 yrs.	HA	For cognitively healthy patients at baseline, who were persistent HA users during follow-up, there was 27% reduced odds of developing dementia, relative to those who were not persistent HA users. Those with dementia at baseline were 54% less likely to be persistent users of HA.	Unclear
Bucholc (2022) USA	4,358	Observational cohort 4 yrs. (mean follow-up)	HA	Reduced MCI likelihood for HA users than individuals with HL who did not use HA. No difference in MCI risk between individuals with HL who used HA and normal hearing individuals.	Yes
Huang et al. (2023)	2,413	Prospective observational	HA	Moderate to severe HL was associated with a higher prevalence of dementia compared to those with normal hearing, and HA use was associated with lower dementia prevalence.	Yes
Sarant (2023) Australia	262	Prospective observational cohort	HA	Better cognitive performance at three-year follow-up for those who used HAs than the comparative group.	Yes
Okano et al. (2024)	36	Case-controlled observational	HA	HA use was associated with stable MMSE-J scores over 12 months compared to matched controls.	Unclear
Cantuaria (2024) Denmark	573,088	Population-based cohort 14 yrs.	HA	Lower dementia risk for individuals with HL who used HAs than individuals who did not use HA.	Yes
Nishiyama et al. (2025)	117	Cross-sectional prospective cohort	HA	In a group of individuals who have never used HAs, HL is related to poorer cognitive scores. However, the negative relationship HL and cognitive function was mitigated by the long-term use of HAs.	Unclear
Voss (2025) Canada	14	Prospective observational	HA	After 12 weeks wearing HAs participants improved on all cognitive test outcomes (visual working memory, executive function and processing speed) except verbal fluency.	Unclear
Vandenbroeke et al. (2025) Belgium	25	Prospective longitudinal	CI	Significant improvements in cognition were observed 1 year after CI, but gains were no longer observed after 4 years.	Unclear

The six trials were small (*N* = 102 or less), with one exception (Lin, 2023) (*N* = 977), and in some cases, these may be underpowered to detect intervention effects. The results of three trials were negative, reporting no difference due to HAs, with the other three equivocal.

The 23 observational and cohort studies showed large methodological differences, studies of both HAs and Cochlear Implants (CIs), with variable sample sizes (*N* = 14 to >500,000) and follow-up (0.5–25 years), different sampling methods and outcome measures (e.g., risk of developing dementia, performance on various cognitive measures). Eleven studies reported positive findings, for example, that HA users had lower dementia risk than non-users (Cantuaria, 2024). One reported negative findings (no differences at all; Dawes et al., 2015); and the results of the other 11 studies were unclear (including all three CI studies).

There are several systematic reviews relevant to this topic. As studies in earlier reviews were incorporated into more recent reviews, only the four most recent reviews are considered here. Two reviews (Carasek et al., 2022; Yeo et al., 2023) examined the role of cochlear implants (CIs)/HAs in modifying cognitive decline and reached positive conclusions; these included mainly observational rather than trial data, with the limitations mentioned above. A systematic review and meta-analysis reported no statistically significant benefit of HAs on cognition (Thosar et al., 2025). Yang et al. (2022) reported no definitive conclusion regarding whether HAs improve cognitive function in individuals without dementia. Machado et al. (2024) reported improvement in quality of life, but not in cognition in individuals provided with HAs.

Given the limited number of randomised controlled trials in this area, there is scope for further high-quality research. However, it is challenging to achieve double-blinding in such trials, and a suitable control group needs careful consideration. It requires a sizeable group of people with hearing loss, who are willing to be potentially randomised into a control group without a hearing intervention and remain as participants over an adequate period of follow-up. As this potentially denies them access to a management option beneficial to their hearing, it poses an ethical dilemma. Future trials could consider innovative study designs; for example, a stepped-wedge design could help mitigate ethical concerns by ensuring that all participants eventually receive the hearing intervention.

## 5 Care and management

Alongside its role as a risk factor, hearing loss commonly co-occurs with dementia. While the potential for cognitive benefit remains uncertain, the value of hearing interventions lies in improving quality of life, enhancing social engagement, and supporting healthy ageing (Tang et al., 2025). HAs remain the standard management option for hearing loss (National Institute for Health and Care Excellence, 2018a), including for people already living with dementia (Ray et al., 2019). The benefits of addressing hearing loss for people living with dementia could include improvements in communication, behavioural and neuropsychiatric dementia symptoms (Mamo et al., 2017; Mamo, 2018; Dawes et al., 2019), and quality of life (Machado et al., 2024). However, people with dementia are less likely to use HAs consistently over time compared to those with intact cognition (Naylor et al., 2022). Inconsistent or discontinued HA use may contribute to an increased rate of cognitive decline, which in turn reduces capacity to maintain HA use, creating a cycle in which untreated hearing loss and cognitive impairment reinforce one another (Naylor et al., 2022).

Effective hearing interventions are not simply a matter of providing a device. Early identification of hearing loss and intervention are critical, as is offering additional support tailored to people with cognitive impairment (Calvert et al., 2025). Recent research has advocated for stronger coordination and integration between audiology and broader healthcare services, to ensure they address the complex needs of people with hearing loss and dementia (Calvert et al., 2025), especially those relying on others to manage their health. For example, in care homes, most people live with either dementia or hearing loss, and many with both, yet HA use is low (RNID, 2018; Prince, 2014; Yamada et al., 2014; Cohen-Mansfield and Taylor, 2004). Reasons for low HA use in care home settings include limited staff awareness of hearing challenges and hearing loss management options, and difficulty in discriminating symptoms and environmental and social factors (Rocks and Ferguson, 2013; Slaughter et al., 2014; Pryce and Gooberman-Hill, 2012). Research suggests that care staff need appropriate training on maintaining HAs and using communication tactics (Crosbie, 2019; Broome and Green, 2026). Despite the known barriers, there are a lack of practical solutions, thus hearing loss remains largely undetected and unmanaged in this population.

To address the lack of specific clinical guidance, interdisciplinary practice recommendations (Littlejohn et al., 2022) and best practice recommendations for the assessment and rehabilitation of hearing loss for people living with dementia (Dawes, 2022) have been published. However, there are currently no known national or international standardised approaches to the assessment and management of hearing loss in this growing population. A qualitative study of UK National Health Service (NHS) audiologists identified recommendations for clinical practice including raising awareness of the importance of hearing care, providing dementia-specific training and practice guidance for audiologists and strengthening post-audiology pathway support, for example mobile clinics within the community (Calvert et al., 2025).

While current dementia pharmacological therapies primarily aim to slow disease progression (National Institute for Health and Care Excellence, 2018b), many non-pharmacological interventions are designed to improve quality of life for people living with dementia (Livingston et al., 2024). Quality of life interventions such as music or reminiscence therapy should ensure that any hearing loss is managed to maximise accessibility and effectiveness. In this context, addressing hearing loss should be viewed as part of broader health management, with benefits for communication, social engagement, quality of life, and the management of dementia-related symptoms.

Given the large number of individuals living with one, or both conditions, it is essential that research and healthcare efforts focus not only on risk factors and mechanisms but also on improving practical care and support for this population. A recently completed James Lind Alliance (JLA) Priority Setting Partnership (PSP) in co-existing dementia and hearing conditions identified and prioritised questions for future research in this area (Heffernan et al., 2025). JLAs bring together people with lived experience and relevant professionals in partnership to identify unanswered research questions that are most important to them, shaping the future research agenda.

The priority questions arising from the PSP spanned diverse topics, from identifying and understanding causal mechanisms through to diagnosis and treatment. However, people living with hearing loss and/or dementia and their care partners have been identified as groups who encounter barriers to accessing clinical services and participating in research (NIHR, 2022; Witham et al., 2020). Barriers to research participation are compounded when people with hearing loss and dementia are also members of additional under-represented communities, such as ethnic minority groups, socio-economically disadvantaged groups, people living in care settings and the Deaf community (NIHR, 2022; Witham et al., 2020; Flower et al., 2025).

**Figure 2:**
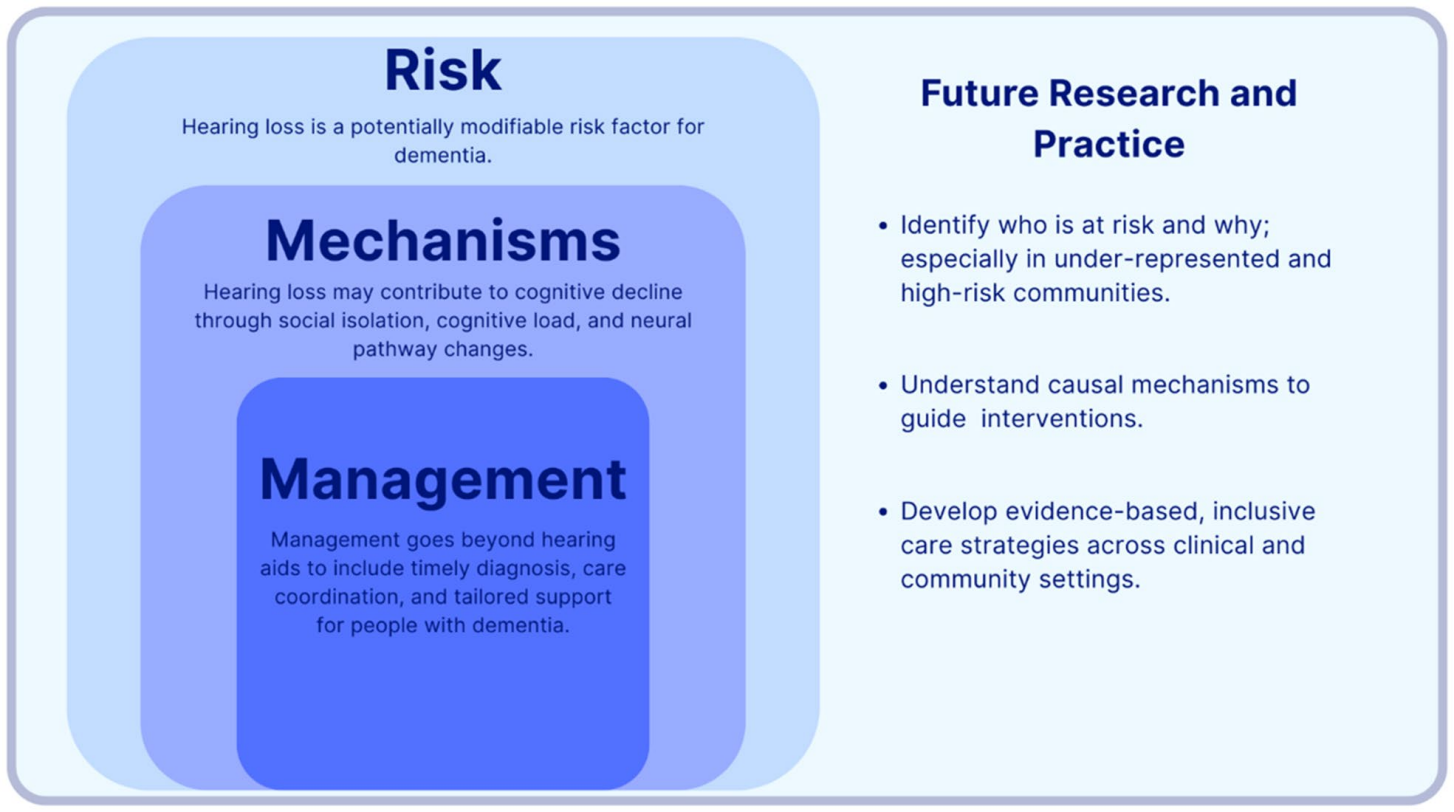
Conceptual pathway to inform future research and practice on hearing loss in dementia.

Greater efforts are needed to ensure that future research in this field is inclusive of under-represented communities. Different populations may have greater risk of one or both conditions and thus require different interventions and enhanced access to health and care services. Studies included in the Lancet Commission are predominantly from high-income countries with limited representation from groups with lower levels of education, and minority ethnic groups (Livingston et al., 2024). The latest Commission report acknowledges that population attributable risk factors are highly swayed by population factors (Livingston et al., 2024). A greater focus on inclusion in future research would help ensure that findings have real-world applications, thereby enhancing the quality of care for these populations and helping reduce health inequalities. Future research should therefore focus on identifying the optimal timing of hearing intervention across the life course, determining which subgroups are most likely to benefit, and clarifying how hearing care can be most effectively integrated into existing dementia care pathways without overburdening patients or services (Figure 2).

Improving public awareness of the benefits of addressing unmanaged hearing loss is one potential component of a public health policy aimed at increasing hearing loss management for all. Integrating hearing loss education for health professionals across the care pathway is essential to ensure that they recognise the early signs of hearing loss and provide appropriate referrals or support at the earliest opportunity. An approach recommended by the World Health Organization is to offer regular hearing screenings for adults to improve early detection of hearing loss to mitigate the long-term costs for patients and health systems more generally (World Health Organization, 2021), whereas UK opinion (Stancel-Lewis et al., 2025) suggests that targeted, risk-stratified hearing screening offers a practical and equitable approach that is consistent with the NHS 10-year health plan for England (Department of Health and Social Care, 2025).

## 6 Discussion

This review highlights the complexity of the association between hearing loss and dementia, while underscoring the uncertainties that remain in determining cause, mechanisms, and the effectiveness of hearing interventions for cognitive decline and dementia risk. Translating primarily epidemiological associations into mechanistic understanding and effective clinical or public health action remains challenging.

The inclusion of hearing loss as a key potentially modifiable mid-life risk factor in successive Lancet Commission reports has been instrumental in raising awareness of the importance of hearing health for dementia risk (Livingston et al., 2017; Stancel-Lewis et al., 2025; Parmar et al., 2025). However, population-attributable fraction (PAF) estimates are model-dependent, context-specific, and rely on assumptions that are not yet fully supported by experimental evidence (Mansournia and Altman, 2018; Ishak et al., 2025). A risk for the field is that an oversimplified interpretation of these estimates may lead to the conclusion that managing hearing loss can completely remove the risk posed by it, eliminating a proportion of dementia cases. Responsible communication is therefore essential to ensure messaging is framed in the context of the available evidence to minimise any negative impacts on unrealistic expectations, help-seeking, and stigma for hearing loss and dementia (British Society of Audiology, 2024; Blustein et al., 2023; Dawes and Munro, 2024).

Evidence shows that the association between dementia and hearing loss is biologically plausible; supported by multiple hypothesised mechanisms. However these may not operate in isolation. Multiple, potentially interactive mechanisms likely link hearing loss with dementia. These include sensory deprivation, increased cognitive load, vascular and neurodegenerative processes, with shared genetic and biological risk factors; all of which are likely to contribute, rather than underly a single unifying pathway. Importantly, the relative contribution of these mechanisms may vary across individuals and across the life course, influenced by factors such as age, sex, cardiovascular health, and timing of the intervention. In particular, mechanisms associated with midlife hearing loss are more consistent with long-term causal pathways, whereas associations observed in later life may increasingly reflect prodromal neurodegenerative processes or shared ageing-related pathology. The recently proposed ‘cause–catalyst–consequence’ framework provides a valuable integrative model for conceptualising these interacting pathways and may help guide future hypothesis-driven research (Homans et al., 2017).

Despite a wealth of associative evidence, we lack substantive experimental evidence for hearing interventions to prevent or meaningfully slow cognitive decline in the general population. Importantly, studies have begun to identify meaningful targeting of hearing intervention and support (Yu et al., 2025). In the meantime, hearing interventions hearing interventions remain clinically valuable for improving communication, social participation, and overall wellbeing. These outcomes are intrinsically important to people living with dementia and their families (Yang et al., 2022; Machado et al., 2024; Tang et al., 2025; National Institute for Health and Care Excellence, 2018a; Ray et al., 2019; Mamo et al., 2017; Mamo, 2018). The ethical and practical challenges of conducting long-term trials in this area suggest that innovative study designs will be required to advance the evidence base.

The comorbidity of hearing loss and dementia is high. While dementia drug therapies primarily aim to slow disease progression, many non-pharmacological interventions focus on improving quality of life, wellbeing and social engagement (Livingston et al., 2024). Failure to identify and manage hearing loss may therefore limit the accessibility and effectiveness of these approaches. In this context, addressing hearing loss should be viewed as an integral component of holistic dementia care, with benefits for communication, participation, behavioural symptoms, and quality of life, even in the absence of disease-modifying effects.

A recurring limitation across the literature is the under-representation of diverse populations, including people from low- and middle-income countries, minority ethnic groups, socioeconomically disadvantaged communities, care home residents, and Deaf communities (Livingston et al., 2024; National Institute for Health and Care Excellence, 2018b; Heffernan et al., 2025). Given the high global prevalence of both hearing loss and dementia, and the substantial variation in access to hearing care, improving inclusivity is essential for ensuring that research findings translate into equitable public health and clinical practice.

The James Lind Alliance Priority Setting Partnership in coexisting dementia and hearing conditions highlights the importance of research questions that matter to people with lived experience, spanning mechanisms, diagnosis, intervention, and service delivery (Dawes, 2022).

## 7 Conclusion

Hearing loss is consistently associated with increased risk of cognitive decline and dementia, although the mechanisms underlying this relationship remain incompletely understood. Current evidence suggests that multiple biological and cognitive pathways likely interact across the life course. Clarifying these pathways and identifying individuals most likely to benefit from intervention will be essential to inform future prevention and management strategies. Progress in this field will depend on interdisciplinary collaboration, inclusive research practices, and a balanced narrative that recognises both the promise and limitations of the evidence to support the role of hearing loss management in dementia prevention and care.
